# Preclinical Evaluation of a Replication-Deficient Intranasal ΔNS1 H5N1 Influenza Vaccine

**DOI:** 10.1371/journal.pone.0005984

**Published:** 2009-06-19

**Authors:** Julia Romanova, Brigitte M. Krenn, Markus Wolschek, Boris Ferko, Ekaterina Romanovskaja-Romanko, Alexander Morokutti, Anna-Polina Shurygina, Sabine Nakowitsch, Tanja Ruthsatz, Bettina Kiefmann, Ulrich König, Michael Bergmann, Monika Sachet, Shobana Balasingam, Alexander Mann, John Oxford, Martin Slais, Oleg Kiselev, Thomas Muster, Andrej Egorov

**Affiliations:** 1 Avir Green Hills Biotechnology AG, Vienna, Austria; 2 Influenza Research Institute, Russian Academy of Medical Sciences, St. Petersburg, Russia; 3 Retroscreen Virology Ltd., Centre for Infectious Diseases, Bart's and the London, Queen Mary's School of Medicine and Dentistry, London, United Kingdom; 4 Division of General Dermatology, Department of Dermatology, Medical University of Vienna, Vienna, Austria; 5 Department of Surgery, University of Vienna Medical School, Vienna, Austria; 6 BioTest Ltd., Konarovice, Czech Republic; Instituto Butantan, Brazil

## Abstract

**Background:**

We developed a novel intranasal influenza vaccine approach that is based on the construction of replication-deficient vaccine viruses that lack the entire NS1 gene (ΔNS1 virus). We previously showed that these viruses undergo abortive replication in the respiratory tract of animals. The local release of type I interferons and other cytokines and chemokines in the upper respiratory tract may have a “self-adjuvant effect”, in turn increasing vaccine immunogenicity. As a result, ΔNS1 viruses elicit strong B- and T- cell mediated immune responses.

**Methodology/Principal Findings:**

We applied this technology to the development of a pandemic H5N1 vaccine candidate. The vaccine virus was constructed by reverse genetics in Vero cells, as a 5∶3 reassortant, encoding four proteins HA, NA, M1, and M2 of the A/Vietnam/1203/04 virus while the remaining genes were derived from IVR-116. The HA cleavage site was modified in a trypsin dependent manner, serving as the second attenuation factor in addition to the deleted NS1 gene. The vaccine candidate was able to grow in the Vero cells that were cultivated in a serum free medium to titers exceeding 8 log_10_ TCID_50/_ml. The vaccine virus was replication deficient in interferon competent cells and did not lead to viral shedding in the vaccinated animals. The studies performed in three animal models confirmed the safety and immunogenicity of the vaccine. Intranasal immunization protected ferrets and mice from being infected with influenza H5 viruses of different clades. In a primate model (*Macaca mulatta*), one dose of vaccine delivered intranasally was sufficient for the induction of antibodies against homologous A/Vietnam/1203/04 and heterologous A/Indonesia/5/05 H5N1 strains.

**Conclusion/Significance:**

Our findings show that intranasal immunization with the replication deficient H5N1 ΔNS1 vaccine candidate is sufficient to induce a protective immune response against H5N1 viruses. This approach might be attractive as an alternative to conventional influenza vaccines. Clinical evaluation of ΔNS1 pandemic and seasonal influenza vaccine candidates are currently in progress.

## Introduction

The increased circulation of highly pathogenic avian influenza viruses in birds with a periodic lethal infection of humans has lasted for more than ten years now. The number of people infected by H5 influenza has already reached 408 in 2009. A very high mortality rate (exceeding 50%) and the appearance of several distinct clades of H5N1 viruses intensify the necessity for an efficient cross-clade protective vaccine to prevent a possible pandemic among the naïve human population.

Clinical trials that have been undertaken with conventional inactivated vaccines that were made from H5N1 viruses indicated that a high dose (up to 90 µg) and double immunization might be essential for an efficient immune response in humans [1]–[4]. The dose sparing as well as cross-neutralizing activity against antigenically different H5N1 strains could be achieved by using immunological adjuvants such as conventional aluminum or newly developed oil-in-water emulsions (MF59, ASO3) [5]–[10]. Two doses of aluminum adjuvanted H5N1 split vaccine elicited a substantial immune response even in naïve children aged 6 months to 9 years, although a higher dose of antigen per child (30 µg) was used than what is used in a seasonal inactivated influenza vaccine [11]. In contrast to subunit and split vaccines, a whole virion inactivated vaccine (WIV) is more immunogenic because of the immune adjuvant effect of viral genomic ssRNA [12], [13]. It was shown that H5N1 WIV that was produced in Vero cells did not require the addition of adjuvant and induced a cross-clade neutralizing antibody response after double immunization with a 7.5–15 µg dose [4]. Unfortunately, WIV may cause a high incidence of adverse events, including local reactions at the site of injection and febrile illness, particularly among children [14], [15].

It is not clear whether the appearance of cross-reactive serum antibodies induced by adjuvanted inactivated vaccines in humans can secure the protection of people against an infection with antigenically distinct H5N1 strains. Respiratory mucosa associated secretory IgA antibodies as well as cytotoxic T lymphocytes (CTL) were shown to be involved in cross-protection [16]–[25]. Several attempts have been made to apply inactivated vaccines intranasally. A combination of intranasally administered inactivated vaccine preparations with mucosal adjuvants, such as cholera toxin B or *Escherichia coli* heat-labile toxin B, could provide cross-protection in mice [26]–[29]. Promising results were obtained in mice with an influenza Virus-Like Particle (VLP) intranasal vaccine eliciting a cross-protective immune response without the addition of an adjuvant after two immunizations [30]. However, in people, the intranasal administration of a virosomal seasonal vaccine with *Escherichia coli* heat-labile toxin was associated with the appearance of Bell's palsy syndrome [31]. Therefore, the development of safe and efficient mucosal adjuvants is an actual issue at hand [32], [33].

Live attenuated vaccine, mimicking a natural infection, is another option for creating broad protection at the mucosal surfaces by an induction of not only systemic but also local IgA, as well as T-cell responses [20], [34], [35]. Only preclinical data are available on the immunogenicity and protective efficacy of H5N1 live vaccine candidates. It was demonstrated that two immunizations with a live attenuated influenza vaccine provide full protection against pulmonary replication and death from a heterologous H5N1 challenge virus in mouse and ferret models [36], [37]. Such vaccines can especially be useful for priming naïve children [38]–[40]. However, vaccine virus replication as well as wheezing syndrome in children 6 to 11 months of age raise some safety concerns [41], [42].

We developed a new type of intranasal influenza vaccine based on the construction of replication-deficient influenza viruses lacking the non-structural protein 1 (ΔNS1 virus). The NS1 protein is considered the major factor antagonizing the innate immune response [43]–[45]. The intranasal administration of ΔNS1 mutant viruses causes the local induction of type I interferons (IFN) in the absence of detectable virus replication [46]. This approach combines the advantage of live attenuated vaccines to induce secretory antibody as well as a cellular immune response without the disadvantage of vaccine virus shedding.

In the present study, we demonstrate that intranasal immunization with an H5N1 ΔNS1 vaccine candidate induces an immune response against the antigenic variants of modern H5N1 viruses in mice, ferrets, and macaques. Moreover, the vaccine was able to elicit protection against heterologous H5 challenge viruses.

## Materials and Methods

### Cells and Viruses

A Vero (WHO-certified) cell line was obtained from the European Collection of Cell Cultures and was adapted and further cultivated at 37°C and 5% CO_2_ in a serum-free Opti-pro medium (Invitrogen) supplemented with 4 mM L-glutamine (Invitrogen).

MDCK cells were cultivated at 37°C and 5% CO_2_ in DMEM medium (Invitrogen) comprising 2% Fetal Bovine Serum (FBS, Invitrogen) and 2 mM L-glutamine.

Human bronchial epithelial 16HBE14o^–^ (HBE) cells (obtained from Dr J. Seipelt, Vienna, Austria) were grown in a minimal essential medium (MEM; Invitrogen) supplemented with 10% FBS and 2 mM L-glutamine. Dishes were coated with 10 µg/ml BSA (Sigma), 30 µg/ml bovine collagen type I (Promocell), and 10 µg/ml human fibronectin (BD Pharmingen) in Ham's F12 medium (HyClone).

All the recombinant viruses that were used in the present study were obtained by reverse genetics solely on Vero cells. The vaccine candidate inherited the HA, NA, and M genes from the H5N1 influenza virus A/Vietnam/1203/04 (A/VN/1203/04). The HA polybasic cleavage site of the H5N1 highly pathogenic strain was replaced by the trypsin specific cleavage site TETR/GLF [47]. The internal protein genes were derived from the IVR-116 vaccine strain distributed by the WHO. IVR-116 is a reassortant that inherited HA and NA genes from influenza A/New Caledonia/20/99 (H1N1), the PB1 gene from A/Texas/1/77 (H3N2), and all other genes from the A/Puerto Rico/8/34 (H1N1) (PR8) virus. Virus IVR-116 was adapted to Vero cells resulting in the appearance of mutations in PA (N10S, P275L, and D682N), NP (S287N), M1 (A146T), and M2 (A27T) proteins. The NS1 open reading frame (ORF) was deleted in the NS segment, as described in Garcia-Sastre et al. [44]. The resulting virus was named VN1203ΔNS1.

Additional influenza A viruses were constructed as 6∶2 reassortants having both the HA with a modified cleavage site, as described above, and the NA of influenza viruses A/VN/1203/04 (H5N1), A/Hong Kong/213/03 (H5N1), and A/Indonesia/05/05 (H5N1) respectively, in combination with all other genes including the complete NS segment of the IVR-116 strain. These viruses were named VN1203, HK213, and IND05, respectively, and were used for challenge experiments in addition to the low pathogenic A/Duck/Singapore-Q/F119/97 (H5N3) (Dk/Sing/97) virus. For the challenge of ferrets, the IND05 FA (ferret adapted) virus was generated by infecting ferrets followed by virus amplification on Vero cells from lung homogenate. No mutations in HA were revealed.

All of the animal studies were approved by the local authorities: the chicken study by the Austrian Federal Ministry of Science and Research; the mouse study by the Russian Institutional Local Ethics Committee; one ferret and the macaque studies by the Institutional Animal Care and Use Committee (IACUC) and the Committee for Animal Protection of the Ministry of Industry and Trade of the Czech Republic; and the ferret challenge study by Retroscreen was conducted in compliance with the UK Home Office Scientific Animals Procedures Act 1986.

### Plasmids and Transfection

cDNAs of H5N1 segments were synthesized (Geneart) based on the sequences derived from the database: A/VN/1203/04 (Accession numbers AY818135 for HA, AY818141 for NA, and AY818144 for M), A/Indonesia/05/05 (EF541394 for HA, and EF541395 for NA), A/Hong Kong/213/03 (AB212054 for HA, and AB212056 for NA).

All synthesized segments were cloned into the bidirectional plasmid pHW2006, a synthetically produced analog of pHW2000 [48]. Just as in pHW2000, it contains a Pol I and Pol II expression cassette for the bidirectional transcription of influenza segments. Plasmids according to the composition of the desired virus were combined and the Vero cells were transfected as described by Kittel et al. [49].

### Virus Propagation and Titration

Seed virus stocks were generated by passaging the transfection supernatant in Vero cells cultivated at 37°C and 5% CO_2_ in an Opti-pro medium supplemented with 4 mM L-glutamine and 5 µg/ml porcine trypsin (Sigma). Infectious virus titers in 50% tissue culture infectious doses (TCID_50_/ml) were determined in Vero cells and calculated according to Reed and Muench.

Influenza virus Dk/Sing/97 was propagated in the allantoic cavity of 9- to 11-day old embryonated hen's eggs at 37°C. Allantoic fluid was collected 48 hours (h) post infection (p.i.).

### Plaque Assay with and without Trypsin

Vero cells were inoculated with serial tenfold dilutions of viruses. After 30 minutes (min) of incubation, the inoculum was removed and the cells were overlaid with 0.6% w/v agar (Sigma) mixed with 50% v/v Opti-pro medium, 4% v/v saturated NaHCO_3_, 6.25% v/v 10× DMEM (Promocell), 1% v/v of 1% DEAE-Dextran, and with or without 5 µg/ml trypsin (Sigma). Plaques were counted after 2 to 3 days incubation at 37°C 5% CO_2_.

### Replication and Induction of Cytokines in Human Macrophages

Peripheral blood mononuclear cells (PBMCs) were obtained by gradient centrifugation with Ficcol-Paque (Farmacia). CD14 positive cells were isolated by immunomagnetic sorting by using the VARIOMACS technique (Miltenyi Biotec GmbH) according to the manufacturer's procedure and cultured in polystyrene tissue culture plates with a hydrophobic surface (Greiner Bio-One). 2×10^6^ cells per 6-well plate were seeded in an RPMI 1640 medium (Invitrogen) supplemented with 10% FCS (HyClone) and 250 U/ml of recombinant human granulocyte-macrophage colony stimulating factor (GM CSF; Berlex) and incubated at 37°C and 5% CO_2_ for 7 days with the addition of 1 ml medium every second day.

On day 7, the macrophages were washed and infected with VN1203ΔNS1 or VN1203 at a multiplicity of infection (moi) of 2 for the determination of the induction of cytokines or moi of 0.001 for the control of virus replication. After an inoculation time of 30 min, the cells were spun down and resuspended in 1 ml RPMI 1640 medium containing 10% FCS and incubated at 37°C and 5% CO_2_. Supernatants were harvested at 24 h (p.i.) and the levels of cytokines (IFN-α, TNF-α, IL-1β, IL-6) were determined by using the Luminex 100 system (Beadlyte Human Multi-Cytokine Detection System 2). Moreover, the amounts of IFN-α were determined by using quantitative cytokine-specific ELISA kits (PBL Biomedical Laboratories) following the manufacturer's instructions. In order to determine the level of virus replication, cells were cultivated in an RPMI medium supplemented with trypsin (5 µg/ml) and the supernatants were collected at 6, 24, 48, and 72 h p.i. and assayed for the presence of infectious virus by a TCID_50_ assay on Vero cells.

### Induction of Interferon in Human Bronchial Epithelial Cells (HBE)

HBE cells were infected with influenza VN1203ΔNS1 or VN1203 viruses at moi 5. Tissue culture supernatants were collected at 6 and 24 h p.i. For control 0.5 µg/ml, poly I:C were transfected to HBE cells by using Lipofectamin 2000 (Invitrogen). The appropriate serial dilutions of these supernatants and of human leukocyte IFN (as a standard; Antigenix America Inc.) in MEM containing L-glutamine and 10% FBS were applied to 96-well plates with A549 cells, stably transfected with a pGL4.17 plasmid (Promega) comprising the firefly luciferase 2 reporter gene under the control of the Mx A promoter [50], [46]. After incubating for 16 h at 37°C and 5% CO_2_, the cells were washed (PBS containing 2 mM EDTA) and treated with 100 µl/well of lysis buffer (8 mM MgCl_2_, 1 mM dithiothreitol, 1 mM EDTA, 15% glycerol, 25 mM Tris-phosphate buffer pH 7.8). 100 µl/well of assay buffer (lysis buffer supplemented with 2 mM ATP and 100 µM D-luciferin sodium salt) were added and luciferase activity was analyzed by using a luminometer (Mediators PHL, Austria) and a 5^th^- parameter logistic standard curve fit (GraphPadPrism software).

### Detection of Serum Antibody Titer by a Hemagglutination Inhibition Assay (HAI)

Sera were diluted 1∶4 with Receptor Destroying Enzyme (RDE; Denka Seiken, Japan) and incubated at 37°C overnight (o.n.). Thereafter, the enzyme was inactivated by heat treatment (56°C for 30 min) and serial two-fold dilutions of sera were prepared in 96-well microtiter plates. 25 µl/well of the standardized antigen (4 hemagglutination units/25 µl) were added. After an incubation period of 60 min at room temperature (RT), 50 µl of 0.5% cRBC or 1% hRBC were added and plates were incubated at RT for 60 minutes.

### Detection of Neutralizing Antibody Titer by a Microneutralization Assay (MNA)

Serial twofold dilutions of RDE-pre-treated sera were prepared in 96-well microtiter plates (Falcon) and 50 µl of a standardized viral suspension (100 TCID_50_/50 µl) were added to each well. After an incubation period of 2 h at 37°C, the Vero cells were added. The plates were incubated for 20 h, washed and acetone fixed. An influenza A virus NP-specific monoclonal antibody (0.125 µg/ml; Chemicon) diluted in a blocking buffer (PBS containing 1% BSA and 0.1% Tween-20) was added for 1 h. Following washing, the bound antibodies were detected by incubating with polyclonal goat anti-mouse IgG HRP conjugate (0.25 µg/ml; KPL). The plates were washed and the substrate (TMB, KPL) was added. The reaction was stopped with 1 N H_2_SO_4_. The average absorption at 450 nm (A_450_) was determined for the control wells of virus-infected (VC) and uninfected (CC) cells and the neutralizing endpoint (NEP) was determined by using a 50% specific signal calculation. 
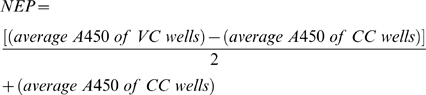
 The endpoint titer was expressed as the reciprocal of the highest dilution of serum with an *A*_450_ value less than NEP.

### Detection of Vaccine Specific Serum IgG and Mucosal IgA

96-well Nunc Maxisorp plates (transparent for serum IgG ELISA and white for mucosal IgA ELISA) were coated with 0.5 µg/ml (100 µl/well) of the recombinant hemagglutinin of the A/VN/1203/04 (H5N1) influenza virus (ferret IgA measurement) (Sinobiological Ltd.) or purified VN1203ΔNS1 virus (mouse IgG measurement) at 4°C o.n. The plates were washed (PBS containing 0.1% Tween-20) and blocked with assay buffer [PBS containing 0.5% I-Block (Tropix) and 0.1% Tween-20]. Serially twofold diluted samples (serum for IgG ELISA or nasal wash for mucosal IgA ELISA) were added and incubated at RT for 1.5 h. On each plate, the reference standards for the respective target antibody, appropriately diluted in the assay buffer, were included. The standard curve for the assessment of H5-specific IgG/IgA was established by utilizing a pool of serum samples (IgG reference standard) or nasal wash samples (IgA reference standard), exhibiting a detectable signal that was determined in a preliminary endpoint ELISA. A 1∶8 dilution of the IgA reference standard was defined as 100 arbitrary units (AU) of H5-specific IgA per ml. After washing, H5-specific IgG or IgA antibodies were detected with goat anti-mouse IgG conjugated to HRP (0.25 µg/ml; Rockland Immunochemicals) or goat anti-ferret IgA conjugated with AP (0.25 µg/ml; Rockland Immunochemicals). The plates were incubated for 1 h, washed again, and TMB (KPL) substrate was added for serum IgG ELISA or Lumi-Phos Plus substrate for mucosal IgA ELISA (Aureon Biosystems). The reaction of the IgG ELISA was stopped with 2 M H_2_SO_4_ and the optical density was determined by using a Biotek photometer (measurement wavelength 450 nm; reference wavelength 630 nm). The luminescence signal in the mucosal IgA ELISA was measured after incubating the plates for 60 min in the dark with a luminometer (Mediators PHL, Austria). H5-specific IgG were presented in log_2_ titer while the concentration of IgA in the individual samples was expressed in AU/ml based on the IgA reference standard calibration curve by the 4-parameter non-linear logistic curve fit (GraphPadPrism software).

In the mucosal samples H5-specific IgA antibodies were normalized. A standard quantitative ELISA was performed by using affinity purified goat anti-ferret IgA (α -chain; 0.25 µg/ml; Novus Biologicals), nasal wash samples, goat anti-ferret IgA (α-chain; 0.25 µg/ml; Rockland Immunochemicals) conjugated with HRP, and TMB substrate (KPL). The IgA concentration in each sample was calculated based on the IgA reference standard curve by a 4-parameter non-linear logistic fit. A dilution of 1∶20 of the IgA reference standard was defined as 100 AU of the total IgA/ml. The final normalized results were expressed in H5-specific AU/100 AU of the total IgA for each individual nasal wash sample.

### Pathogenicity and Infectivity Study in Chickens

The VN1203ΔNS1 vaccine strain virus was administered intravenously (*i.v.*) to a group of eight 5–6 week old White Leghorn SPF chickens at a dose of 7.1 log_10_ TCID_50_/animal in 0.2 ml. In parallel, two other groups of chickens were treated intranasally (*i.n.*) with 0.1 ml of the same virus preparation (6.8 log_10_ TCID_50_/animal) or with PBS. The animals were observed daily for clinical signs and death over a period of 14 days. On day 3, post administration oropharyngeal and cloacal swabs were collected from each chicken and virus replication was assayed by a TCID_50_ assay on Vero cells.

### Protection Efficacy Study in Mice

Groups of 6–8 week old outbred female mice were immunized *i.n.* under narcosis once or twice with 50 µl of the VN1203ΔNS1 virus at a dose of 5.3 log_10_ TCID_50_/animal, 3 weeks apart. The control group was treated with PBS. Three weeks post each immunization, 6 animals from each group were bled and sera were analyzed by HAI and ELISA. The remaining animals treated with the vaccine strain or PBS were challenged under narcosis (6 mice per virus) with 50 µl of VN1203 (3.3 log_10_ TCID_50_/animal), HK213 (3.2 log_10_ TCID_50_/animal), IND05 (2.2 log_10_ TCID_50_/animal), or Dk/Sing/97 (4.2 log_10_ TCID_50_/animal) viruses. On day 3 and 5 post challenge, 6 mice from each group were euthanized; the lung and nasal tissues were collected and a 10% w/v tissue homogenate was prepared. The viral load was determined by a TCID_50_ assay on Vero cells for VN1203, HK213, and IND05 or on MDCK cells for Dk/Sing/97.

### Pathogenicity and Immunogenicity in Ferrets

A group of 22 ferrets (11 males and 11 females) (Biotest, Czech) received two intranasal doses each 7.8 log_10_ TCID_50_/animal of the VN1203ΔNS1 virus in a volume of 0.5 ml via a spray device, 4 weeks apart. The virus was purified and formulated in SPGN buffer (6% w/v sucrose, 3.8 mM KH_2_PO_4_, 7.2 mM K_2_HPO_4_, 4.9 mM L-glutamate, and 75 mM NaCl). At the same time, the control group (n = 16) was immunized *i.n.* with the same amount of SPGN. The animals were examined on a daily basis up to 3 weeks post the second immunization. Sera were collected before the first and second immunization as well as 3 weeks after the second immunization. Four days p.i., 10 animals of the immunized and 6 animals of the mock treated group were sacrificed, tissues were collected (lung, nasal mucosa, brain, kidney, and intestinal tissue) and 10% w/v tissue homogenates were investigated for the viral load by titration on Vero cells. Sera were processed by HAI and MNA.

### Protection Efficacy in Ferrets

This experiment was performed by Retroscreen Virology Ltd., UK. Seven month old seronegative male ferrets were housed in three groups under conventional conditions in floor pens. Anesthetized ferrets (light anesthesia was induced via the inhalation of isoflurane) were immunized intranasally either once (7 animals) or twice (8 animals, with an interval of 18 days) with 8.1 log_10_ TCID_50_/animal of the VN1203ΔNS1 virus (tissue culture supernatant) or with PBS (6 animals). 14 days after the final immunization, the anesthetized animals were challenged intranasally with 6.3 log_10_ TCID_50_/animal of the reassortant virus IND05 FA. The body weight and clinical symptoms e.g. nasal discharge, sneezing, dyspnea, the level of activity as well as mean maximum and mean sum of the inflammatory cell counts in nasal washes were assessed from the 1^st^ to 7^th^ day.

### Collection of Blood and Nasal Washings from Ferrets

1 ml PBS was instilled into each nostril of the anesthetized animal by using a displacement pipette. The ferret was then turned over with its nose resting just over the collection container (Petri-dish). The nostrils were gently tickled by using a pipette tip to instigate sneezing and the expulsion of nasal mucus and nasal wash, which were collected into a Petri-dish and supplemented with 1 ml of media. Nasal washes were collected before immunization, 14 days p.i. and 1, 3, 5, and 7 days post challenge. The nasal wash fluids were frozen at −80°C.

Blood was collected by piercing the shaved and disinfected upper tail area with a syringe needle. Blood was processed to serum and stored at −20°C.

### Evaluation of the Pathogenicity and Immunogenicity in Macaques

The VN1203ΔNS1 virus was administered *i.n.* (7.8 log_10_ TCID_50_/animal) once as a spray in Rhesus macaques (*Macaca mulatta*) ranging in age from 2 to 3 years (Biotest Ltd., Czech Republic). The animals were monitored for clinical signs by daily observations and weekly body weight measurements. In addition, standard hematological and plasma biochemical analyses were performed. Blood samples were collected before immunization and 4 weeks p.i. Nasal washing samples were collected before and 2 days p.i. in order to evaluate the vaccine virus shedding by TCID_50_ titration on Vero cells and to determine the level of cytokines.

### Detection of Cytokines in Macaque Nasal Washings

The level of cytokines in the nasal washings was measured with the Luminex 100 system (Upstate, Temecula, CA).

## Results

### Generation of H5N1 Reassortants

The vaccine candidate VN1203ΔNS1 was designed as a 5∶3 reassortant, inheriting the HA, NA, and M genes from the H5N1 isolate A/VN/1203/04 and the remaining genes from the IVR-116 vaccine strain (see Materials and Methods). The NS fragment was modified by the complete deletion of the NS1 ORF. This virus was rescued entirely from cDNA clones by the co-transfection of a set of eight plasmids into Vero cells. Plasmids encoding genes of avian origin were constructed based on the sequence data obtained from the GeneBank. The polybasic cleavage site of HA was replaced by the sequence TETR/GLF, which was found to be genetically more stable in birds than the sequence (RETR/GLF) that is typical for avirulent H5 viruses and that is widely used for H5N1 vaccine development [47].

The resulting vaccine candidate possessed several attenuating factors, including the modification of HA and NS genes in combination with the replacement of the majority of the avian genes by that of the IVR-116 virus.

By a similar approach, several 6∶2 reassortants, namely VN1203, HK213, and IND05 with functional NS1 gene and modified HA, were generated. These viruses were intended to be replication competent in *vivo* and suitable for challenge experiments in animals.

For the HAI and the MNAs, 6∶2 reassortants VN1203 and IND05 were constructed comprising an additional mutation (S223N) in the HA, as described by Hoffman et al. [51]. The introduced mutation improved HAI performance of the VN1203 virus with chicken red blood cells (cRBC) reached the level of sensitivity attained with horse red blood cells (hRBC), but did not have any effect on the IND05 virus. Therefore, the assay with the IND05 virus was additionally performed with hRBC.

### VN1203ΔNS1 Virus Grows to High Titers in Vero Cells

Rescued virus VN1203ΔNS1 was able to grow in Vero cells to a titer of 8.5 log_10_ TCID_50_/ml. No mutations were detected in the avian HA, NA, or M genes after 8 passages in the Vero cells. The antigenic properties of the VN1203ΔNS1 virus were indistinguishable from the reference strains (performed by Medical Research Council, London, UK; data not shown).

In order to monitor the HA modification, virus replication in the presence and absence of trypsin was investigated in Vero cells by a plaque assay. No plaques were noticed in the absence of trypsin for the VN1203ΔNS1 virus in contrast to the clear plaques observed in cells cultivated with trypsin (data not shown).

### VN1203ΔNS1 Virus Triggers Innate Immune Response in HBE Cells and Human Macrophages

**Figure 1:**
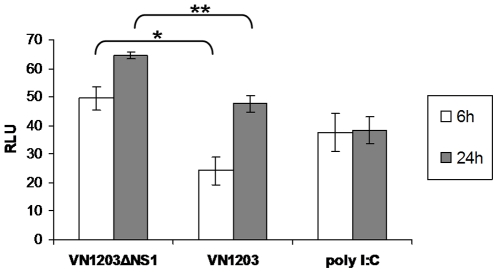
Induction of IFN in human bronchial epithelial (HBE) cells. HBE cells were infected with VN1203ΔNS1 or VN1203 at moi 5. At 6 and 24 h p.i. supernatants were collected. The level of secreted IFN was determined by a bioassay based on the A549 cell line that was stably transfected with a luciferase reporter gene under the control of the MxA promoter. As a control, HBE cells were transfected with 0.5 µg/ml poly I:C, and supernatants were treated like these of the infected cells. Relative light units (RLU) represent the measured luciferase activity. The data from the most representative experiment are shown. * indicates p<0.03, ** p<0.02 determined by a Student's t-test.

**Figure 2:**
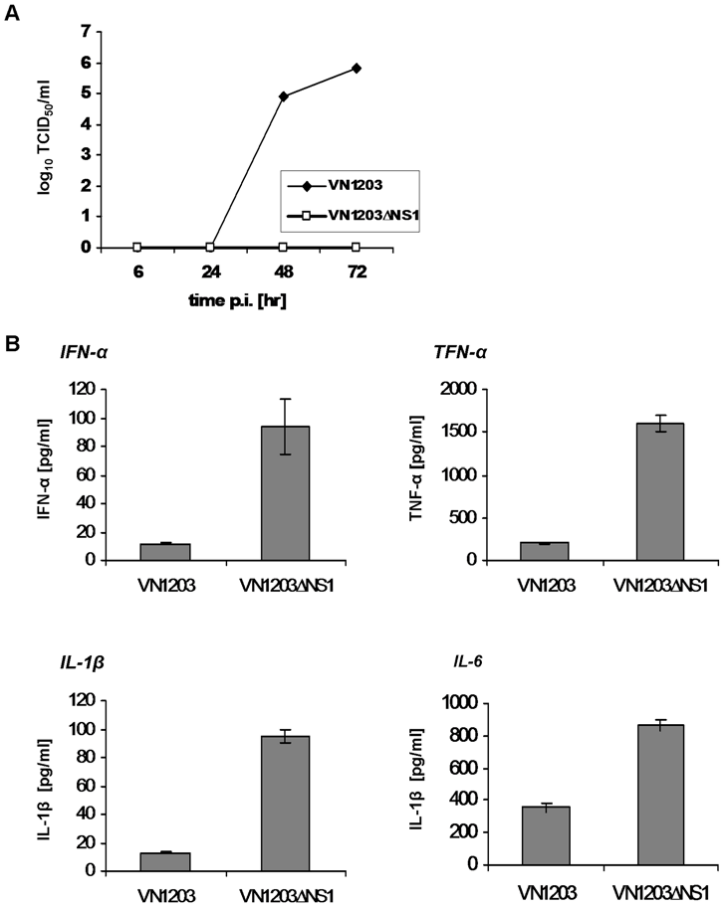
Viral replication and induction of cytokines in human macrophages. (A) Human macrophages were infected with VN1203ΔNS1 or VN1203 at a moi of 0.001. The level of viral replication was determined at different time points by a TCID_50_ assay on Vero cells. (B) Seven day old macrophages were infected with VN1203ΔNS1 or VN1203 at a moi of 2. The supernatants of the infected cells were harvested 24 h p.i and assayed for IFN-α, TNF-α, IL-1β, and IL-6. The experiment was repeated three times. The data of one representative experiment are presented as a mean of two measurements. The mock value is subtracted.

Influenza viruses encoding functional NS1 protein are able to antagonize the cytokine response of infected cells, whereas ΔNS1 mutant viruses induce an elevated level of type I IFNs and other pro-inflammatory cytokines [45], [46]. We used human bronchial epithelial cells (HBE) and macrophages in order to evaluate the innate immune response that is triggered by a VN1203ΔNS1 virus infection. The type I/III IFN response in HBE cells was studied by utilizing the MxA-driven luciferase reporter bioassay (Fig. 1). At time points 6 and 24 h p.i., the vaccine candidate induced significantly (p<0.03) higher increases of luciferase signals compared to a virus containing the full size NS1 gene. The signal induced by the transfected poly I:C was used as a control. The level of pro-inflammatory cytokines (IFN-α, TNF-α, IL-6, and IL-1β) induced by the VN1203ΔNS1 virus in macrophages was up to tenfold higher when compared to the virus with a full size NS1 gene (Fig. 2B). The ΔNS1 virus could not replicate in macrophages in contrast to the analogous virus containing the unaltered NS1 gene, which replicated to max titers of approx. 6 log_10_ TCID_50_/ml (Fig. 2A). These results confirmed the replication deficient phenotype of the VN1203ΔNS1 virus in the IFN competent cells associated with the enhanced triggering of the innate immune system.

### Pathogenicity of VN1203ΔNS1 Virus in Chickens

In order to estimate the level of the attenuation of the VN1203ΔNS1 vaccine candidate in chickens, five to six week old white leghorn SPF chickens (eight per group) were inoculated *i.v.* or *i.n.* with 7.1 or 6.8 log_10_ TCID_50_/animal of VN1203ΔNS1 respectively. No signs of illness or pathological lesions were detected in any of the infected birds. The analysis of the chicken cloacal or oropharyngeal samples taken three days post *i.v.* or *i.n.* infection revealed no infectious virus in the samples, in turn verifying the replication-deficient phenotype of this virus (results not shown).

### VN1203ΔNS1 Virus Provides Cross-reactive Protection in Mice

**Figure 3:**
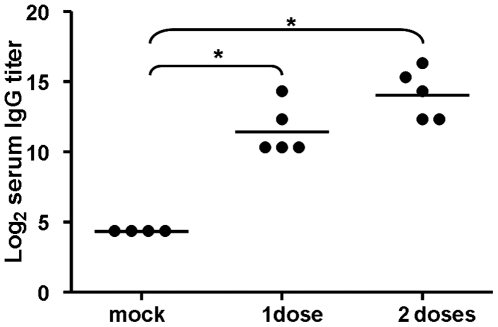
Determination of virus-specific mouse serum IgG titers. Mice were immunized once or twice with 5.3 log_10_ TCID_50_/animal of the VN1204ΔNS1 virus. The control mice were treated with PBS. Serum was collected three weeks p.i. and the level of IgG was determined by ELISA. The serum titers of the individual mice (symbols) expressed as log_2_ and the GMTs (horizontal line) for each group of mice are shown. * indicates p<0.0001 determined by a Student's t-test.

To determine whether the *i.n.* immunization of mice with the VN1203ΔNS1 virus provides protection against homologous or heterologous challenge, the animals were immunized *i.n.* once or twice with 5.3 log_10_ TCID_50_/animal of VN1203ΔNS1 virus. Two days p.i., four animals were sacrificed for the determination of the infectious viral progeny in mouse organs. No viral growth was detected in the nasal, lung, brain, or spleen tissues (data not shown). Sera taken three weeks p.i. were analyzed by HAI and ELISA. HAI titers (made with cRBC) were low (data not shown). However, substantial serum IgG response was found by ELISA after the first immunization (GMT = 2940) and significantly increased after boosting immunization (GMT = 17828) (Fig. 3).

Three weeks after the first or second immunization, the mice were challenged with replicating viral reassortants belonging to H5N1 clade 1 (VN1203, HK213) or clade 2.2 (IND05) as well as with virus Dk/Sing/97 (H5N3). All the viruses were able to grow in the lungs of naïve mice in the range of 4.0–5.0 log_10_ TCID_50_/ml (Table 1). Single intranasal immunization provided substantial protection for the mice, which was reflected in the accelerated virus clearance from the lungs. Statistically significant titer reduction of the challenge viruses was detected on day 3 after infection compared to the control group (p<0.05). On day 5, the levels of the viral load in the lungs of the majority of animals were undetectable, irrespective of the challenge virus. After two immunizations, the virus VN1203ΔNS1 provided nearly full protection from the infection with the challenge viruses belonging to different clades.

**Table 1:** Replication of challenge viruses in mice immunized with the VN1203ΔNS1 virus.

Treatment Group	Challenge Virus	3 d post challenge		5 d post challenge	
		No. of Animals Protected/Total No.^a^	Mean Virus Titer log_10_ b	No. of Animals Protected/ Total No.^a^	Mean Virus Titer log_10_ b
**1 Dose**	VN1203	2/6	2.6±0.5	6/6	<1.5
	HK213	3/6	3.0±0.6	5/6	1.6±0.4
	IND05	1/6	2.9±0.3	5/6	1.6±0.4
	Dk/Sing/97	2/6	2.0±0.4	6/6	<1.5
**2 Doses**	VN1203	6/6	<1.5	6/6	<1.5
	HK213	6/6	<1.5	6/6	<1.5
	IND5	6/6	<1.5	6/6	<1.5
	Dk/Sing/97	6/6	<1.5	6/6	<1.5
**PBS**	VN1203	0/6	4.9±0.2	0/6	4.4±0.2
	HK213	0/6	5.2±0.2	0/6	4.5±0.3
	IND05	0/6	4.0±0.1	0/6	3.7±0.3
	Dk/Sing/97	0/6	4.1±0.3	0/6	4.5±0.3

a Mice with lung virus load <1.5 log_10_ TCID_50_/ml were classified as being protected.

b The virus titers are expressed as the mean log_10_ TCID_50_/ml±standard error (S.E.) from 6 animals. The limit of virus detection was 1.5 log_10_ TCID_50_/ml. Tissues where no virus was detected were defined as 1.5 log_10_ TCID_50_/ml for the calculation of the mean titer.

Mice (6 animals per group) were administered *i.n.* one or two doses of the VN1203ΔNS1 virus (5.3 log_10_ TCID_50_/animal) three weeks apart and were challenged with homologous VN1203 (3.3 log_10_ TCID_50_/animal), or heterologous HK213 (3.2 log_10_ TCID_50_/animal), IND05 (2.2 log_10_ TCID_50_/animal) and Dk/Sing/97 (4.2 log_10_ TCID_50_/animal) viruses three weeks post each immunization. The decrease of the mean virus titers measured in the vaccinated group compared to the PBS treated group was significant in all the groups as determined by the Mann-Whitney U-test (p<0.05).

### Attenuation, Immunogenicity, and Protective Efficacy in Ferrets

**Figure 4:**
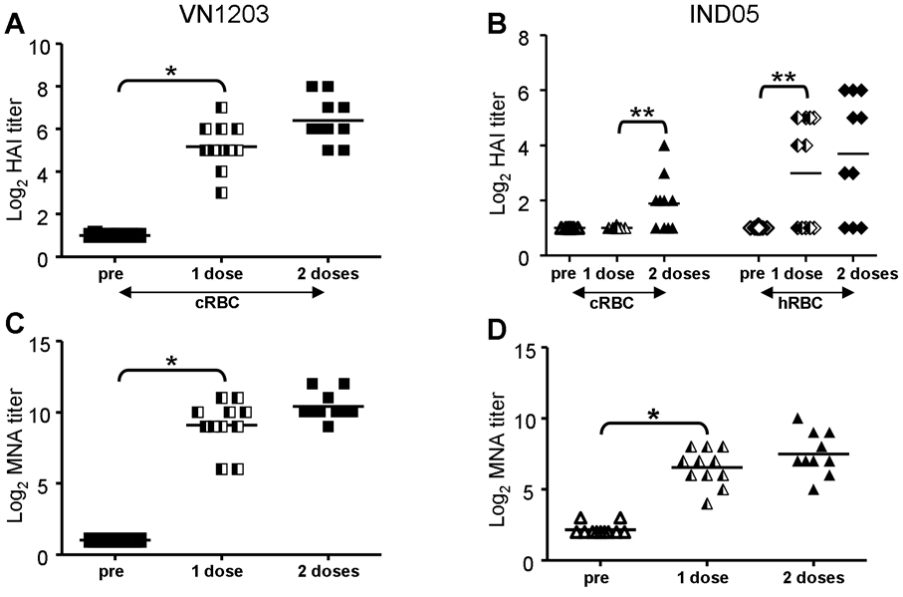
Serum HAI and neutralizing (MNA) antibody titers after single or double *i.n*. immunization of ferrets. Ferrets received two *i.n.* doses of 7.8 log_10_ TCID_50_/animal of VN1203ΔNS1 virus, four weeks apart. Sera were collected 29 days after the 1^st^ and then 21 days after the 2^nd^ immunization and were analyzed for homologous (VN1203) and heterologous (IND05) HAI antibodies (A, B) with cRBC and hRBC or neutralizing (MNA; C, D) antibodies. The titers of the individual ferrets (symbols) and the mean titers (horizontal line) are shown. * indicates p<0.0001, and ** p<0.05 determined by a Student's t-test. An undetectable HAI antibody titer was assigned a value of <8. An undetectable neutralizing antibody titer was assigned a value of <16.

To obtain more detailed information about the safety and immunogenic potential of the VN1203ΔNS1 virus, a repeated dose toxicity study was conducted in ferrets. The animals were administered two *i.n.* doses (7.8 log_10_ TCID_50_/animal) of the VN1203ΔNS1 virus, four weeks apart. In life monitoring performed during seven weeks after the first inoculation indicated no major differences in clinical signs, body weight, or temperature between the treated and control groups (data not shown). No vaccine virus shedding was observed in the nasal washes taken on day three (data not shown). The examination of the viral load in organs taken on day three, post first immunization, revealed no infectious virus in the lung, brain, (three sections including the cerebrum, cerebellum, and pons), intestine, or kidney tissues. Infectious virus was isolated in 2 of the 10 animals (at traceable quantities) only from homogenized nasal turbinates, which most likely represent the residual particles from the high viral inoculation load. The serum antibody response was determined after the 1^st^ and 2^nd^ immunizations by HAI and MNA (Fig. 4). A single vaccination induced an increase in antibody titers in the HAI test (GMT = 36, cRBC) against the homologous VN1203 antigen. The second vaccination doubled the titers (GMT = 84). HAI (hRBC) antibody response to the heterologous IND05 antigen was detected only in one-half of the animals. An MNA test revealed a statistically significant increase in antibody titers after single immunization against homologous (GMT = 545) and heterologous (GMT = 93) viruses followed by a further increase after the second immunization, GMT = 1351 and GMT = 181, respectively.

**Figure 5:**
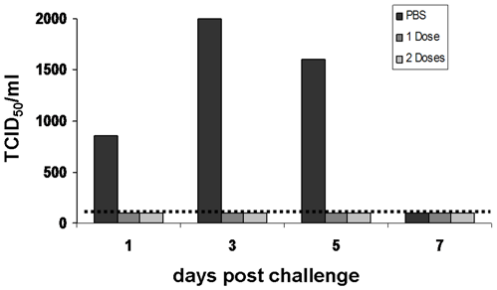
Replication of heterologous challenge virus in immunized ferrets. Ferrets that received either PBS or one or two doses of 8.1 log_10_ TCID_50_/animal of the VN1203ΔNS1 virus were challenged with 6.3 log_10_ TCID_50_/animal of IND05 FA virus two weeks post the last immunization. Nasal samples were collected at the indicated time points and level of infectious virus was determined by the titration on Vero cells. The lower limit of detection is 2 log_10_ TCID_50_/ml indicated by the horizontal dashed line.

**Figure 6:**
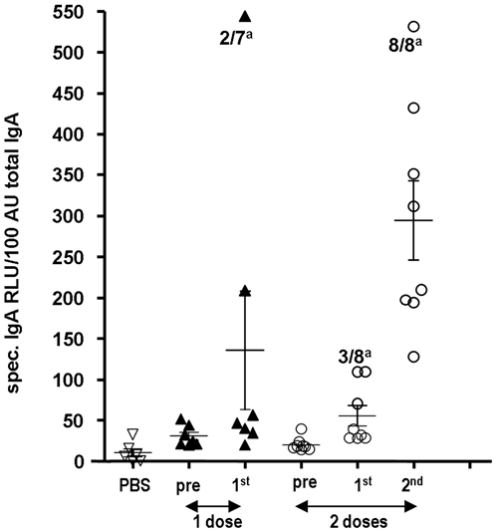
Detection of vaccine virus-specific IgA in the nasal wash samples of immunized ferrets. Ferrets were immunized either with PBS once (6 animals) or with the vaccine virus VN1203ΔNS1 once (7 animals) or twice (8 animals) with a dose of 8.1 TCID_50_/animal. Nasal wash samples were collected prior to immunization (pre) and two weeks after each immunization. Individual vaccine virus specific IgA levels were normalized based on the total IgA content of each sample and expressed as vaccine virus-specific IgA RLU/100 AU (arbitrary units) of total IgA. The means (horizontal lines) and standard errors of mean (vertical lines) are presented. Animals with a 2.5-fold increase in IgA titer following immunization were considered as responders. ^a^No. of responders/total no. of immunized animals.

In a separate experiment, protective immunity against a heterologous strain was tested in animals that were immunized *i.n.* with 8.1 log_10_ TCID_50_/animal of VN1203ΔNS1 once or twice at an interval of 18 days. As in the previous study, serum antibody HAI or MNA titers to the heterologous IND05 strain were detected only after double immunization (results not shown). Ferrets were challenged with the IND05 FA virus taken in a dose of 6.3 log_10_ TCID_50_/animal. Although the challenge virus did not elicit significant symptom scores, its replication in the respiratory tract of ferrets reached a peak of 3.3 log_10_ TCID_50_/ml on day three, post challenge, in the PBS control group (Fig. 5). In the vaccine groups, single and double immunization equally brought the challenge virus to undetectable levels in the nasal washings at any time point after the challenge. This protection at least partially could be attributed to a local IgA immune response. As shown in Fig. 6, H5 specific IgA antibodies in the nasal washes were detected in two of the seven animals after a single immunization, although all of the vaccinated animals responded after the second dose of the vaccine.

### Attenuation and Immunogenicity of the VN1203ΔNS1 Virus in Rhesus Macaques

Since nonhuman primates were shown to be a suitable model for the evaluation of H5N1 influenza pathogenesis [52], next we checked the performance of the VN1203ΔNS1 virus in Rhesus macaques focusing on attenuation and immunogenicity. Adult macaques (2.5–4 years old) were immunized *i.n.* with 7.8 log_10_ TCID_50_/animal of virus without narcosis in order to limit virus application only to the upper part of the respiratory tract mimicking immunization in humans. Vaccination did not provoke any clinical manifestation in the macaques such as fever or respiratory symptoms, confirming the safety of the vaccine in the primate model.

The replication deficient phenotype of the VN1203ΔNS1 virus was corroborated by the absence of virus isolation from the nasal washings taken on day 2 and 4, p.i. (data not shown).

Despite of the lack of active viral replication, the elevated levels of IL-1β, IL-2, IL-6, IL-8, IL-4, IL-12p70, IFNγ, TNFα, and GM CSF cytokines were detected in vaccinated, but not control, macaque nasal lavage fluids collected 2 days post immunization, although statistically significant increase was shown only for IL-1β, IFNγ, TNFα, and GM CSF cytokines (Fig. S1). The spectrum of induced cytokines indicated the possible involvement of epithelial as well as immune competent cells in the generation of antiviral innate immune response similar to the wild type influenza virus infection in humans [53].

**Figure 7:**
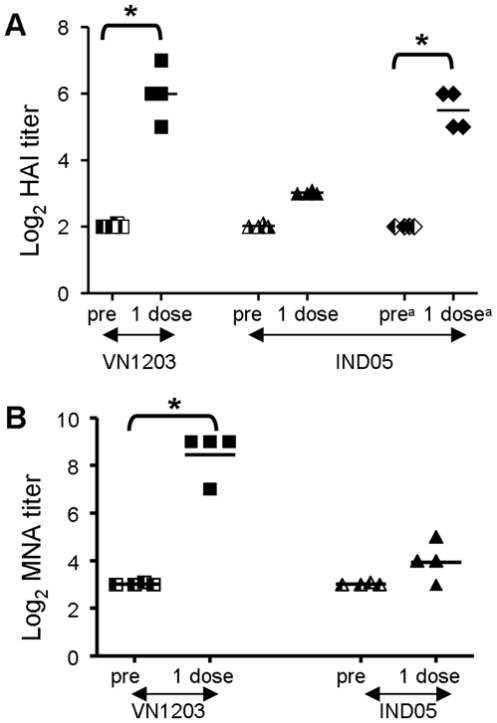
Serum antibody responses after the single *i.n*. immunization of adult *Rhesus macaques*. A group of four macaques was immunized *i.n.* with VN1203ΔNS1 at a dose of 7.8 log_10_ TCID_50_/animal. Sera were collected four weeks p.i. and HAI with homologous VN1203 (cRBC) or heterologous IND05 (cRBC and hRBC indicated by ^a^) antigens and MNA titers were performed. Undetectable HAI antibody titer was assigned a value of <8. An undetectable neutralizing antibody titer was assigned a value of <16. * indicates p<0.05 determined by the Mann-Whitney U-test.

A single immunization with the VN1203ΔNS1 virus induced a significant increase in HAI (cRBC) (GMT = 64) and neutralizing antibodies (GMT = 362) against homologous virus VN1203. Moreover, these antibodies persisted during a 6-month observation period and titers dropped only 2 times (data not shown). An increase against the heterologous IND05 virus was detected by HAI assay only with hRBC (GMT = 45) but not in MNA (GMT = 16) (Fig. 7). Therefore, intranasal immunization of non-human primates with the strain VN1203ΔNS1 was safe, did not provoke virus shedding, and induced a specific antibody response.

## Discussion

The increased incidence of human infection with highly pathogenic H5N1 avian influenza viruses with high mortality rate serves as an alarm for the global society. The problem does not only concern the efficacy of the licensed types of influenza vaccines, but also the capacity of the world industry to produce sufficient doses of any influenza vaccine for stock piling. A pandemic vaccine must be capable of inducing protection against hypothetical viruses that so far have not spread in the human population, but that may arise. This difficult situation evokes several new directions in influenza vaccine research and development. Limitations in the availability of embryonated chicken eggs stimulate efforts to use continuous cell lines for influenza vaccine production [54], [55]. The necessity for antigen sparing and the problem of cross-reactivity has sparked an intensive search for new immunological adjuvants [10], [56]. Live attenuated intranasal and VLP vaccines that are capable of inducing secretory IgA and T-cell immunity are considered as a possible solution for obtaining broader protection against subtype specific antigenic variants. Mucosal vaccines might be especially useful during pandemic periods due to the simplicity of their application.

We developed a new type of intranasal influenza vaccine that aims at combining the strong sides of live and inactivated influenza vaccines. This approach is based on the production of over-attenuated, replication deficient vaccine viruses with deleted NS1 ORF and, therefore, lacking antagonistic effects on the innate immune system. Innate immunity is the first line of defense against viruses and microbes and acts nearly immediately to limit the early proliferation and spread of infectious agents. This includes the activation of phagocytic and antigen-presenting cells, such as dendritic cells (DCs) and macrophages, as well as the initiation of inflammatory responses through the release of a variety of cytokines, chemokines, and antimicrobial factors, such as IFNs and defensins [57], [58]. The NS1 protein is the main factor that enables influenza viruses to invade the host and successfully replicate on the background of the down regulated IFN system [59], [60]. This function of the NS1 protein is required at the first hours after infection, and determines the early and abundant mode of its synthesis [59]. The impairment of NS1 protein function leads to abortive replication due to the induction and activation of a variety of antiviral proteins such as protein kinase R (PKR) and 2′,5′-oligoadenilate synthetase (OAS) molecules, leading to an antiviral state in infected and neighboring cells [61] [43], [46]. Therefore, immunization with the ΔNS1 vaccine could be considered similar to vaccination with virus-like particles, since it does not induce vaccine virus shedding [30]. At the same time, ΔNS1 mutant viruses are infectious for nasal respiratory cells and can evoke synthesis and the presentation of viral antigens similar to live attenuated vaccines [46].

Important, that the method of attenuation of viruses by deleting interferon antagonist must be suitable for different age groups of people and people having innate immune deficiencies. It should be noted that innate immune system is already active in newborn children [62]. The autosomal recessive form of human complete Stat-1 deficiency is an extremely rare disorder, so far reported in three unrelated patients [63]. Such children usually develop disseminated bacillus Calmette-Gue'rin (BCG) after vaccination and subsequently died of viral illnesses. Another cellular defense mechanism against influenza mediated by dsRNA activated protein kinase (PKR) was also shown to be matured during foetal development (reviewed by Garcia et al. [64]. To our knowledge PKR deficiency related pathology was not described in people. In general, innate immune deficiency should be suspected in children with severe mycobacterial or viral diseases [63]. Such children should be excluded from any vaccination programs. In elderly, immunosenescence renders vaccination to be less effective. Aging leads to a decline in the response to infection by both the innate and adaptive immune systems. Therefore, ΔNS1 vaccine approach has to be evaluated more carefully in old people and patients with secondary immune deficiencies.

We applied the ΔNS1 technology to construct H5N1 vaccine candidates. Such a vaccine might be an appropriate alternative to conventional vaccines in case of a pandemic, due to simplicity of mucosal immunization. In addition, in contrast to live influenza vaccines, the absence of replication and shedding may decrease the risk of adverse events and vaccine virus spread in immunologically naïve population, particularly children.

The vaccine virus was created as a 5∶3 reassortant containing a combination of three avian virus genes (HA with a modified cleavage site, NA and M) with the other 5 genes from the IVR-116 vaccine virus strain, which was adapted for growth in Vero cells and modified by the deletion of NS1 ORF. The inclusion of the avian M gene and, therefore, the encoding of the M1 and M2 proteins of emerging H5N1 pandemic strains may bring important protective epitopes for a T- and B- cell immune response, which might not be present in old influenza human isolates. At the same time, the 5∶3 genome composition of the vaccine reassortant should not affect the safety level of the vaccine since modified NS and HA genes are present as main contributors of attenuation.

We demonstrated that H5N1 ΔNS1 deletion mutant viruses can be successfully produced in IFN deficient Vero cells, which are qualified for human use, reaching titers exceeding 8.0 log_10_/ml, in turn making the production of such vaccines feasible and comparable to production in eggs. We evaluated the ΔNS1 H5N1 vaccine candidate in three animal models, namely mouse, ferret, and non-human primate. None of the animals developed any serious adverse events or lung pathology, thus reinforcing the safety profile of the vaccine virus. No infectious virus was isolated from distant studied organs or the nasal washings of inoculated animals, confirming the replication deficient phenotype of the vaccine candidate. Two cases of virus isolation from destructed nasal tissue samples could be explained by vaccine virus survival after the application of the high viral dose (7.5 log_10_/ferret) in stabilizing formulation.

We confirmed for the H5N1 vaccine candidate the previous observations that ΔNS1 mutant viruses intensively trigger the innate immune response of infected cells. We demonstrated that the infection of human bronchial epithelial cells and macrophages caused the profound release of type I IFNs and other pro-inflammatory cytokines analogous to ΔNS1 mutants of other subtypes [45]. Intranasal immunization of macaques showed increased levels of the pro-inflammatory cytokines and chemokines in the nasal mucosa. The spectrum of detected cytokines indicates that both infected epithelial cells as well as immune competent cells are most probably involved in cytokine production. It was reported that type I IFNs as well as IL-1β, IL-12, IL-18, and GM-CSF can exhibit mucosal adjuvant activity for the induction of serum IgG and mucosal IgA antibody responses when nasally administered with protein antigens [65]. Therefore, we hypothesize that ΔNS1 vaccines can stimulate an adaptive immune response without adding immunological adjuvants (self adjuvant effect).

Studies in animals demonstrated that despite a replication deficient phenotype, the H5N1 ΔNS1 vaccine virus was immunogenic in mice, ferrets, and monkeys. Significant serum antibody response to a homologous strain was detected by different methods in all animals. However, in mice the IgG titers were detected after the first immunization only in ELISA but not by the functional HAI assay with cRBC. Therefore, we tested the protection efficacy in mice by using four different H5N1 and H5N3 challenge viruses of various clades that are capable of replicating in mouse lungs efficiently. Surprisingly, the rate of protection was nearly equal against all the challenge viruses irrespective of their antigenic properties. One immunization was sufficient for the accelerated clearance of the infection from mouse lungs, whereas complete protection was achieved after two immunizations. The lack of HAI antibodies in mice remains to be explained since in other animal models, the functional assays revealed a substantial antibody response. In ferrets and macaques, the first immunization was sufficient to detect HAI antibody titers to VN1203 antigen. Moreover, in macaques, a comparable antibody response to both viral clades was obtained after a single immunization when hRBC were used. A challenge experiment in ferrets revealed that a single immunization was effective for preventing an infection with a heterologous strain despite the lack of HAI antibodies in some animals. Thus, the appearance of neutralizing antibodies to IND05 detected in MNA in turn correlated better with the protection of ferrets than HAI antibodies. Besides for systemic cross-neutralizing antibodies, the induction of H5 specific mucosal IgA that was detected in some ferrets after the first immunization as well as the T-cell response may contribute to the protection of ferrets against a heterologous virus. Unfortunately, the method for measurement for the T-cell response in ferrets is not yet established.

Our results demonstrate the potency of the H5N1 ΔNS1 vaccine candidate for evoking a cross-reactive H5N1 specific immune response in three animal models. However, the protection efficacy of this vaccine candidate remains to be confirmed with non-modified highly pathogenic H5N1 viruses. An experiment in macaques is currently in progress.

The ΔNS1 vaccine approach is applicable for any of the influenza subtypes. The safety and immunogenicity of the influenza A H1N1 vaccine strain lacking the NS1 gene was studied in a first-in-man clinical trial, showing the full safety profile and a high immune response in adult volunteers (paper in preparation). The Phase I clinical evaluation of the intranasal H5N1 pandemic ΔNS1 vaccine candidate is ongoing.

## Supporting Information

Figure S1 Level of cytokines in nasal washings of macaques. A group of four macaques was immunized i.n. with VN1203ΔNS1 at a dose of 7.8 log10 TCID50/animal. Nasal washings were collected 2 days p.i. Cytokines were measured by using the Luminex 100 system (Beadlyte Human Multi-Cytokine Detection System 2)
(0.28 MB TIF)
