# Supplementary figures and images for: Preclinical Evaluation of a Replication-Deficient Intranasal ΔNS1 H5N1 Influenza Vaccine
Source: PLoS One. 2009 Jun 19;4(6):e5984. doi: 10.1371/journal.pone.0005984 (PMC2694350; doi:10.1371/journal.pone.0005984)

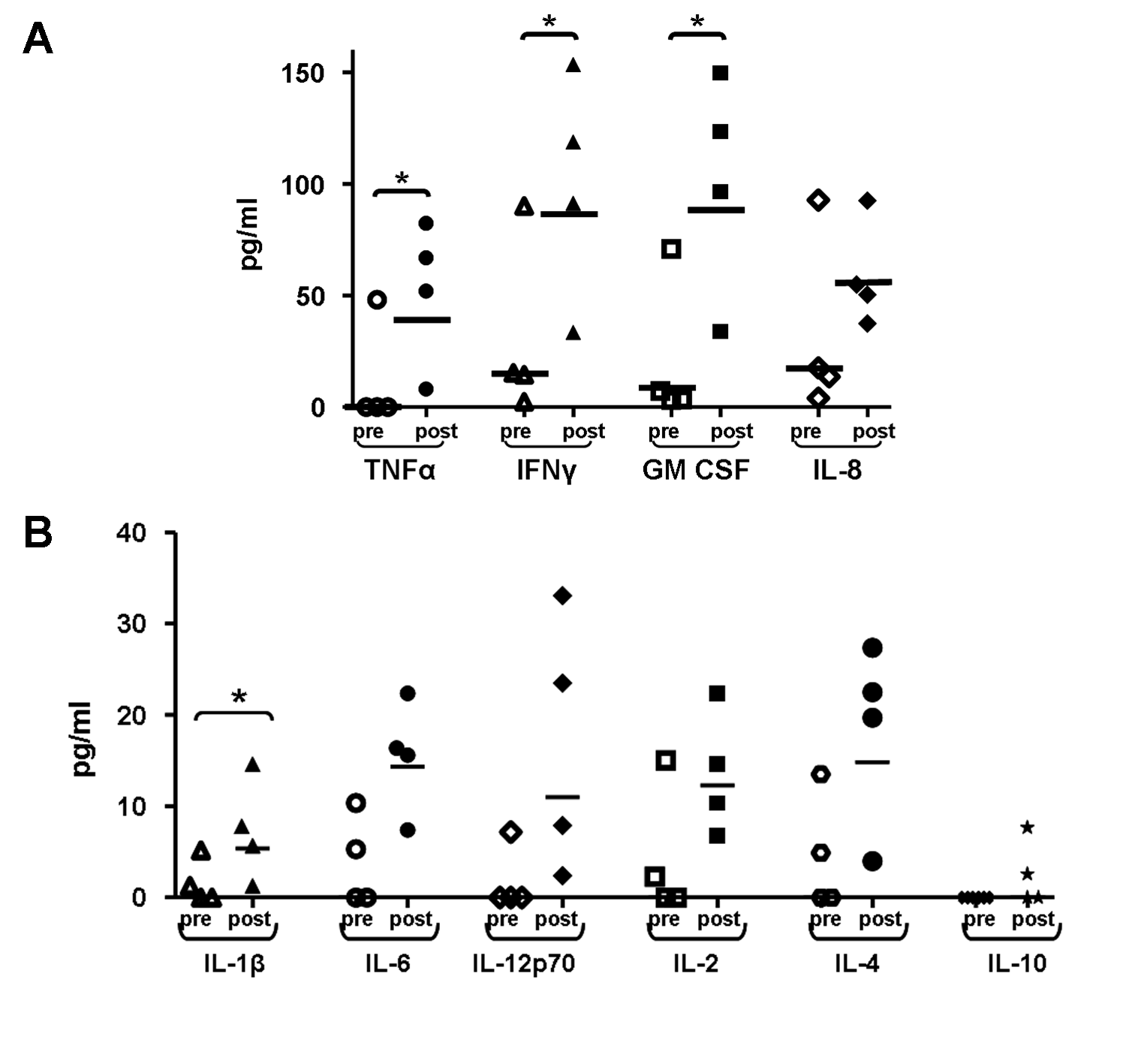

Supplement: Figure S1 — Level of cytokines in nasal washings of macaques. A group of four macaques was immunized i.n. with VN1203ΔNS1 at a dose of 7.8 log10 TCID50/animal. Nasal washings were collected 2 days p.i. Cytokines were measured by using the Luminex 100 system (Beadlyte Human Multi-Cytokine Detection System 2) (0.28 MB TIF) [file pone.0005984.s001.tif]
